# Death from Chlorofom

**Published:** 1850-01

**Authors:** Samuel Solly

**Affiliations:** Surgeon to St. Thomas’ Hospital


### DEATH FROM CHLOROFOM.

COMMUNICATED BY SAMUEL SOLLY, ESQ., SURGEON TO ST. THOMAS' HOSPITAL.

John Shorter, aged 48, a porter, known to Mr. Solly for some time as a very active messenger, habits intemperate, but

Page(s) missing

